# Prevalence of Consensual Male–Male Sex and Sexual Violence, and Associations with HIV in South Africa: A Population-Based Cross-Sectional Study

**DOI:** 10.1371/journal.pmed.1001472

**Published:** 2013-06-18

**Authors:** Kristin L. Dunkle, Rachel K. Jewkes, Daniel W. Murdock, Yandisa Sikweyiya, Robert Morrell

**Affiliations:** 1 Department of Behavioral Sciences and Health Education, Rollins School of Public Health, Emory University, Atlanta, Georgia, United States of America; 2 Center for AIDS Research, Rollins School of Public Health, Emory University, Atlanta, Georgia, United States of America; 3 Gender and Health Research Unit, Medical Research Council of South Africa, Pretoria, South Africa; 4 Programme for the Enhancement of Research Capacity, University of Cape Town, Cape Town, South Africa; University of California, San Francisco, United States of America

## Abstract

**Background:**

In sub-Saharan Africa the population prevalence of men who have sex with men (MSM) is unknown, as is the population prevalence of male-on-male sexual violence, and whether male-on-male sexual violence may relate to HIV risk. This paper describes lifetime prevalence of consensual male–male sexual behavior and male-on-male sexual violence (victimization and perpetration) in two South African provinces, socio-demographic factors associated with these experiences, and associations with HIV serostatus.

**Methods and Findings:**

In a cross-sectional study conducted in 2008, men aged 18–49 y from randomly selected households in the Eastern Cape and KwaZulu-Natal provinces provided anonymous survey data and dried blood spots for HIV serostatus assessment. Interviews were completed in 1,737 of 2,298 (75.6%) of enumerated and eligible households. From these households, 1,705 men (97.1%) provided data on lifetime history of same-sex experiences, and 1,220 (70.2%) also provided dried blood spots for HIV testing. 5.4% (*n* = 92) of participants reported a lifetime history of any consensual sexual activity with another man; 9.6% (*n* = 164) reported any sexual victimization by a man, and 3.0% (*n* = 51) reported perpetrating sexual violence against another man. 85.0% (*n* = 79) of men with a history of consensual sex with men reported having a current female partner, and 27.7% (*n* = 26) reported having a current male partner. Of the latter, 80.6% (*n* = 21/26) also reported having a female partner. Men reporting a history of consensual male–male sexual behavior are more likely to have been a victim of male-on-male sexual violence (adjusted odds ratio [aOR] = 7.24; 95% CI 4.26–12.3), and to have perpetrated sexual violence against another man (aOR = 3.10; 95% CI 1.22–7.90). Men reporting consensual oral/anal sex with a man were more likely to be HIV+ than men with no such history (aOR = 3.11; 95% CI 1.24–7.80). Men who had raped a man were more likely to be HIV+ than non-perpetrators (aOR = 3.58; 95% CI 1.17–10.9).

**Conclusions:**

In this sample, one in 20 men (5.4%) reported lifetime consensual sexual contact with a man, while about one in ten (9.6%) reported experience of male-on-male sexual violence victimization. Men who reported having had sex with men were more likely to be HIV+, as were men who reported perpetrating sexual violence towards other men. Whilst there was no direct measure of male–female concurrency (having overlapping sexual relationships with men and women), the data suggest that this may have been common. These findings suggest that HIV prevention messages regarding male–male sex in South Africa should be mainstreamed with prevention messages for the general population, and sexual health interventions and HIV prevention interventions for South African men should explicitly address male-on-male sexual violence.

*Please see later in the article for the Editors' Summary*

## Abstract 2

Using a method that offered complete privacy to participants, Rachel Jewkes and colleagues conducted a survey among South African men about their lifetime same-sex experiences.

*Please see later in the article for the Editors' Summary*

## Editors' Summary

**Background:**

AIDS first emerged in the early 1980s among gay men living in the US, but it soon became clear that AIDS also infects heterosexual men and women. Now, three decades on, globally, 34 million people (two-thirds of whom live in sub-Saharan Africa and half of whom are women) are infected with HIV, the virus that causes AIDS, and 2.5 million people become infected every year. HIV is most often spread by having unprotected sex with an infected partner, and most sexual transmission of HIV now occurs during heterosexual sex. However, 5%–10% of all new HIV infections still occur in men who have sex with men (MSM; homosexual, bisexual, and transgender men, and heterosexual men who sometimes have consensual sex with men). Moreover, in the concentrated HIV epidemics of high-income countries (epidemics in which the prevalence of HIV infection is more than 5% in at-risk populations such as sex workers but less than 1% in the general population), male-to-male sexual contact remains the most important transmission route, and MSM often have a higher prevalence of HIV infection than heterosexual men.

**Why Was This Study Done?:**

By contrast to high-income countries, HIV epidemics in sub-Saharan Africa are generalized—the prevalence of HIV infection is 1% or more in the general population. Because male-to-male sexual behavior is criminalized in many African countries and because homosexuality is widely stigmatized, little is known about the prevalence of consensual male–male sexual behavior in sub-Saharan Africa. This information and a better understanding of male–female sexual concurrency (having overlapping sexual relationships with men and women) and of how male-to-male transmission contributes to generalized HIV epidemics is needed to inform the design of HIV prevention strategies for use in sub-Saharan Africa. In addition, very little is known about male-on-male sexual violence. Such violence is potentially important to study because we know that male-on-female violence is associated with increased HIV risk for both victims and perpetrators. In this cross-sectional study (an investigation that measures population characteristics at a single time point), the researchers use data from a population-based household survey to investigate the lifetime prevalence of consensual male–male sexual behavior and male-on-male sexual violence (victimization and perpetration) among men in South Africa and the association of these experiences with HIV infection.

**What Did the Researchers Do and Find?:**

About 1,700 adult men from randomly selected households in the Eastern Cape and KwaZulu-Natal provinces of South Africa self-completed a survey that included questions about their lifetime history of same-sex experiences using audio-enhanced personal digital assistants, a data collection method that provided a totally private and anonymous environment for the disclosure of illegal and stigmatized behavior; 1,220 of them also provided dried blood spots for HIV testing. Ninety-two men (5.4% of the participants) reported consensual sexual activity (for example, anal or oral sex) with another man at some time during their life; 9.6% of the men reported that they had been forced to have sex with another man (sexual victimization), and 3% reported that they had perpetrated sexual violence against another man. Most of the men who reported consensual sex with men, including those with current male partners, reported that they had a current female partner. Men with a history of consensual male–male sexual behavior were more likely to have been a victim or perpetrator of male-on-male sexual violence than men without a history of such experiences. Finally, men who reported consensual oral or anal sex with a man were more likely to be HIV+ than men without such a history, and perpetrators of male-on-male sexual violence were more likely to be HIV+ than non-perpetrators.

**What Do These Findings Mean?:**

These findings provide new information about male–male sexual behaviors, male-on-male sexual violence, male–female concurrency, and HIV prevalence among men in two South African provinces. The precision of these findings is likely to be affected by the small numbers of men reporting a history of consensual male–male sexual behavior and of male-on-male sexual violence. Importantly, because the study was cross-sectional, these findings cannot indicate whether the association between consensual male–male sexual behaviors and increased risk of male-on-male sexual violence is causal. Moreover, these findings may not be generalizable to other regions of South Africa or to other African countries. Nevertheless, these findings suggest that information about the risks of male–male sexual behaviors should be included in HIV prevention strategies targeted at the general population in South Africa and that HIV prevention interventions for South African men should explicitly address male-on-male sexual violence. Similar HIV prevention strategies may also be suitable for other African countries, but are likely to succeed only in countries that have, like South Africa, decriminalized consensual homosexual behavior.

**Additional Information:**

Please access these websites via the online version of this summary at http://dx.doi.org/10.1371/journal.pmed.1001472.

- This study is further discussed in a *PLOS Medicine* Perspective by Jerome Singh
- Information is available from the US National Institute of Allergy and Infectious Diseases on HIV infection and AIDS
- NAM/aidsmap provides basic information about HIV/AIDS, including summaries of recent research findings on HIV care and treatment
- Information is available from Avert, an international AIDS charity, on many aspects of HIV/AIDS, including information on HIV and men who have sex with men, on HIV prevention, and on AIDS in Africa (in English and Spanish)
- The US Centers for Disease Control and Prevention also has information about HIV/AIDS among men who have sex with men (in English and Spanish)
- Patient stories about living with HIV/AIDS are available through Avert; the charity website Healthtalkonline also provides personal stories about living with HIV

## Introduction

In the concentrated HIV epidemics of high-income countries, men who have sex with men (MSM) have a higher HIV prevalence than heterosexual men [1]–[5]. In the generalized epidemics of sub-Saharan Africa, MSM have historically been marginalized in HIV research. The population prevalence of consensual male–male sexual behavior has not been well described, nor have associations between consensual male–male sexual behavior and HIV been described in population-based studies. Given the high HIV prevalence among MSM in other settings, understanding of the prevalence of MSM, male–female sexual concurrency (having overlapping sexual relationships with men and women), and the contribution of male–male transmission to African epidemics is critical for understanding how best to approach HIV prevention in this setting.

Male–male sexual behavior is criminalized in many African countries [6], and homosexuality is widely stigmatized, creating difficulty in research on African MSM [7]. There is evidence that African MSM are vulnerable to HIV, other sexually transmitted infections, and sexual violence [8]–[13], and to a range of high-risk behaviors including substance abuse [8], unprotected anal intercourse [8],[9],[11],[14], and transactional sex [9],[12],[15]. However, existing research has tended to use convenience, venue-based, snowball, or respondent-driven sampling—sampling methods that are generally biased towards men who are part of social and sexual networks of self-identified MSM at the expense of closeted or isolated MSM.

MSM in Africa, as elsewhere, are diverse in identities, attractions, and sexual behavior. Research suggests that many African MSM do not self-identify as gay or homosexual [9],[16], and that male–female concurrency is common [12],[15]. While some studies report high levels of sexual violence between MSM [12], male-on-male sexual violence has only rarely been examined among African men in the general population [17]. Understanding the prevalence of consensual male–male sexual behavior and sexual violence between men in South Africa, and the links of these with HIV, is important for contextualizing the contribution of both consensual male–male sex and male-on-male sexual violence to a generalized epidemic, and to understanding the sexual health needs of all men and their male and female sexual partners.

The analyses presented here address the following questions: What is the population prevalence of consensual male–male sexual behavior among South African men? What is the population prevalence of male-on-male sexual violence victimization and perpetration? What socio-demographic factors are associated with these experiences? Are these experiences associated with a greater risk of prevalent HIV infection? To answer these questions, we draw on data from a population-based household survey of adult men in two South African provinces that included information on lifetime history of consensual sex with men, history of male-on-male sexual violence victimization and perpetration, and HIV serostatus.

## Methods

A cross-sectional household survey was conducted in 2008 in three adjoining districts of the Eastern Cape and KwaZulu-Natal provinces of South Africa, spanning rural areas, commercial farmland, towns, and a major city. Detailed methods are described elsewhere [18]. A population-based sample of men aged 18–49 y was identified using a multistage proportionate sampling design. Census enumeration areas (EAs) were the primary sampling units, and the sample was drawn from those in the 2001 census by Statistics South Africa, stratified by district and proportionate to population size. Within each EA, we mapped all households, randomly sampled 20 households, enumerated the eligible men who slept within the house the previous night, and randomly selected one. There was no replacement of households without an eligible man.

Among 222 sampled EAs, two (0.9%) had no human dwellings. In the remaining 220, one (0.45%) was excluded because gatekeepers refused access, and four (1.8%) were excluded because no homes with eligible men were identified following multiple visits at different times of day. We sampled a total of 4,473 visiting points. Of these, 822 (18.4%) could not be rostered for eligibility after a minimum of three attempts at contact. Among the remaining 3,651 visiting points, 1,353 (37.1%) were found to contain no eligible man, while 2,298 (62.9%) contained at least one eligible man. We thus estimated a total eligible population of 2,815 men in our sampling frame. Of this estimated population, 27% could not be contacted (estimated *n* = 760), 10.5% (*n* = 296) refused to participate, 0.7% (*n* = 21) agreed to complete interviews but then either withdrew or failed to provide any usable data, and 61.7% (*n* = 1,737) completed the questionnaire. Interviews were thus completed in 215 of 220 eligible EAs (97.7%), and in 1,737 of 2,298 enumerated and eligible households (75.6%). From these households, 1,705 men (97.1%) provided data on lifetime history of same-sex experiences, and 1,220 (70.2%) also provided dried blood spots for HIV testing.

Participants self-completed a survey using audio-enhanced personal digital assistants (APDAs). The text of each question and associated answer choices were presented on the APDA screen, while an accompanying voice recording read the question and answers aloud. The questions could be read, or listened to, in isiXhosa or isiZulu and English. All questions had fixed multiple choice answers (including a “refuse to answer” option). Answer choices were pre-translated and cross-validated across all three languages by multilingual study staff with native fluency in isiXhosa and isiZulu. Interested readers may contact the corresponding author, R. K. J., for further details on the questionnaire. Participants listened to questions through headphones, and answered by tapping their answer choice with a stylus.

This environment provided complete privacy for respondents. Fieldworkers were nearby during questionnaire completion so they could assist respondents if requested, but interviews were otherwise private. Questions included demographics (including self-identified race because it is strongly associated with HIV prevalence), socioeconomic status (SES), and detailed sexual histories. Participants were also asked for a finger-prick blood spot that was dried for HIV testing. To guarantee total anonymity, no identifying information was collected nor retained after an interview was completed.

### Sexual Attraction and Consensual Male–Male Sexual Activity

Sexual attraction was asked as “Which gender attracts you sexually?” Response options included “women,” “men,” “both,” and “unsure.” Lifetime history of consensual male–male sexual activity was assessed by asking: “Have you ever had sex or done something sexual with a man? By sex we mean: Anal sex: where a man sticks his penis in another man's anus; Oral sex: when a man sticks his penis in another man's mouth; Masturbation: when one or both men play with each others' sex organs; Thigh sex: when a man has sex by putting his penis between another man's closed thighs.” If yes, we asked individual yes/no questions about each named act “done with a man because you wanted to.” Participants reporting a history of consensual sex with men were asked whether they had a current male partner. Men who indicated any consensual sex with a man in response to these questions are referred to as MSM in this paper; those with only victim or perpetrator experiences are not.

### Male-on-Male Sexual Violence

To measure the lifetime history of male-on-male sexual violence victimization we asked “Did a man ever persuade or force you to have sex when you did not want to?” If yes, we asked, “Which of the following acts took place with a man when you did not want to?” with yes/no response categories for anal, oral, masturbation, and thigh sex. Lifetime history of sexual violence perpetration was queried as, “Have you ever done anything sexual with a boy or man when he didn't consent or you forced him?” and “Have you ever done anything sexual with a boy or man where you put your penis in his mouth or anus when he didn't consent or you forced him?” Male-on-male rape victimization and perpetration were defined in analyses as any nonconsensual oral and/or anal penetration.

### HIV Serostatus

Dried blood spots were tested for HIV with a screening ELISA (Genscreen, Bio-Rad), and positive results were confirmed with a second ELISA (Vironostika, bioMérieux).

71.6% (*n* = 1,220/1,705) of men included in these analyses provided blood for HIV testing. Men who declined did not differ significantly from those who did in age, race, circumcision, history of consensual male–male sexual activity, or perpetration of male-on-male sexual violence. However, men who had completed secondary education were significantly less likely to provide blood (66.6% [459/689] versus 75.1% [756/1,007], *p* = 0.0003), and men reporting sexual violence victimization were significantly more likely to provide blood (80.9% [1,211/1,688] versus 70.8% [1,080/1,526], *p* = 0.01).

### Ethics

Ethics approval was granted by the Medical Research Council's Ethics Committee. Participants signed informed consent separately for interviews and dried blood spots. An incentive of ZAR 25 (∼US$3.50) was offered for each component, giving a total of ZAR 50 for both. The APDAs ensured that participant answers were entirely anonymous, and we retained no identifying information on any participant. This was necessary to protect participants who reported illegal activities from possible repercussions. It also meant that HIV results could not be given to the participants. Free HIV testing is widely available from government clinics in South Africa, and all participants were advised to learn their status.

### Data Analysis

The sample was self-weighting. Questionnaire data were linked to HIV data using anonymous codes. Analyses were performed using Stata 12.0 and accounted for the two-stage sampling structure, with stratification by district and data clustered in EAs. No imputation methods were used to replace missing data.

The distribution of socio-demographic characteristics, sexual attraction, sexual behaviors, and experiences with violence were summarized as percentages (or means), using standard methods for estimating confidence intervals from complex multistage sample surveys (Taylor linearization). Pearson's chi-square was used to test two-way associations between categorical variables and consensual male–male sexual behavior, male-on-male sexual violence victimization, or male-on-male sexual violence perpetration. Socio-demographic correlates of consensual male–male sexual experience, male-on-male sexual violence victimization, and male-on-male sexual violence perpetration were described using maximum likelihood multivariable logistic regression. All variables significant at *p*<0.20 in bivariate analyses were tested for inclusion in the models; variables significant at *p*<0.05 were retained in the final multivariable regression models.

Associations between the three experiences of interest (consensual male–male sexual experience, male-on-male sexual violence victimization, and male-on-male sexual violence perpetration) and HIV status were also described using maximum likelihood multivariable logistic regression. All theoretically relevant variables and all variables shown to be significantly associated with any of the three experiences of interest were tested as potential confounding variables, and were retained in the final models if they altered the point estimate for any association between male–male contact and HIV by 10% or more [19]. Hunger in the household was the only variable that met these criteria. All models were adjusted for age, race, and circumcision, which have previously been shown to predict prevalent HIV infection in this sample [20].

## Results

### Prevalence and Socio-Demographic Correlates of Consensual Male–Male Sexual Behavior

Overall, 5.4% (*n* = 94; 95% CI 4.4–6.6) of men reported any consensual sexual activity with another man: 2.8% reported mutual masturbation (*n* = 47/1,705); 1.8% (*n* = 30/1,705), thigh sex; 1.8% (*n* = 30/1,705), anal sex; 1.7% (*n* = 29/1,705), oral sex; and 1.7% (*n* = 28/1,705) did not specify. Some of the men reported multiple forms of sex. In all, 2.6% (*n* = 44; 95% CI 1.9–3.5) reported consensual oral or anal sex with another man, representing 48% of men reporting any consensual male–male sexual behavior.

As shown in Table 1, there were no significant differences in the prevalence of consensual male–male sexual behavior by race, age, education, or recent employment. Consensual male–male sexual behavior was less often reported by men with higherSES; it was also more often reported by men with hunger in their households, but this was not significant in multivariable modeling. Almost all men in this study had had sex with a woman, including 98.9% of MSM. There were no differences in consensual male–male sexual behavior among men who had ever married women, who had fathered children, or who had a current female partner. MSM were more likely to report having a current female partner than a current male partner (85.0% versus 27.7%), and a majority of MSM with a current male partner also reported having a current female partner (80.8%; *n* = 21/26). A slight majority of MSM (64.9%) reported being sexually attracted to women, while 21.3% reported being sexually attracted to men or to both men and women, and 13.8% were unsure. Self-reported sexual attraction was significantly associated with consensual male–male sexual behavior, with an adjusted odds ratio (aOR) of 3.37 (95% CI 1.81–6.27) for men attracted to men or both men and women, and an aOR of 4.18 (95% CI 2.05–8.51) for men who replied “not sure.”

**Table 1:** Socio-demographic characteristics, sexual behavior, and sexual attraction by lifetime history of any consensual sexual activity with a man (MSM).

Category	Characteristic	Ever Engaged in Consensual Male–Male Sexual Behavior (*n* = 94)		Never Engaged in Consensual Male–Male Sexual Behavior (*n* = 1,592)		*p*-Value	Odds Ratio (95% CI)	aOR^a^ (95% CI)
		Percent (Number)	95% CI	Percent (Number)	95% CI			
**Demographics**	**Racial group**							
	Non-black African	86.2 (81)	(77.7–97.8)	84.9 (1,350)	(80.1–88.7)	0.74	Ref	
	Black African	13.8 (13)	(8.2–22.3)	15.2 (241)	(11.3–19.9)		1.11 (0.60–2.04)	
	**Age group**							
	18–24 y	47.9 (45)	(37.3–58.6)	51.9 (826)	(48.8–54.9)	0.47	Ref	
	25 y and older	52.1 (49)	(41.4–62.7)	48.1 (766)	(45.1–51.2)		1.17 (0.77–1.78)	
	**Secondary education**	36.3 (33)	(26.9–46.7)	40.8 (647)	(37.8–43.9)	0.37	0.83 (0.53–1.28)	
**SES**	Mean SES score (range: 6–19)	11.6 (n/a)	(11.1–12.2)	12.6 (n/a)	(12.5–12.8)	**0.001**	**0.86 (0.79–0.94)**	**0.83 (0.84–0.99)**
	Hunger in household	63.5 (54)	(52.6–73.2)	51.1 (748)	(48.0–54.2)	**0.03**	**1.67 (1.06–2.62)**	
	Employed	62.8 (59)	(51.8–72.6)	54.2 (857)	(51.3–57.0)	0.13	1.44 (0.93–2.22)	
**Family and relationships**	Ever had sex with a woman	98.9 (93)	(92.7–99.9)	95.3 (1,512)	(94.0–96.3)	0.10	4.61 (0.63–33.6)	
	Ever married a woman	54.8 (51)	(45.1–64.3)	45.5 (714)	(42.6–48.4)	0.43	1.46 (0.96–2.24)	
	Ever fathered children	54.8 (51)	(44.0–65.3)	45.3 (707)	(42.6–48.1)	0.09	1.47 (0.96–2.23)	
	Current female partner	85.0 (79)	(75.9–91.0)	86.9 (1,373)	(84.9–88.7)	0.60	0.85 (0.47–1.53)	
	Current male partner	27.7 (26)	(20.1–36.8)	n/a	n/a	n/a	n/a	
	Current male and female partners (both)	22.6 (21)	(15.5–31.7)	n/a	n/a	n/a	n/a	
**Sexual attraction**	Women	64.9 (61)	(54.8–73.8)	87.4 (1,384)	(85.7–89.1)	**<0.001**	Ref	Ref
	Men/both	21.3 (20)	(14.1–30.7)	6.8 (108)	(5.7–8.2)		**4.20 (2.44–7.22)**	**3.37 (1.81–6.27)**
	Not sure	13.8 (13)	(8.3–22.2)	5.8 (91)	(4.6–7.1)		**3.24 (1.72–6.11)**	**4.18 (2.05–8.51)**
**Sexual violence**	Male SV victim	34.4 (32)	(25.7–44.4)	8.1 (129)	(6.8–9.7)	**<0.001**	**5.92 (3.72–9.43)**	**6.92 (4.11–11.6)**
	Male SV perpetrator	10.9 (10)	(6.1–18.5)	2.6 (40)	(1.9–3.5)	**<0.001**	**4.65 (2.24–9.63)**	**2.47 (1.04–5.87)**

Values in bold are significant at *p*<0.05.

a Only correlates significant at *p*<0.05 are included.

n/a, not applicable; Ref, reference value; SV, sexual violence.

### Prevalence and Socio-Demographic Correlates of Male-on-Male Sexual Violence Victimization

Overall, 9.5% (*n* = 162; 95% CI 8.0–11.0) of men in this sample reported male-on-male sexual violence victimization, while 3.3% (*n* = 50; 95% CI 2.5–4.1) had been orally or anally raped by another man. As shown in Table 2, male-on-male sexual violence victimization was more often reported by men who were black African, who were aged 25 y or older, or who had fathered children. Victimization was less often reported by men with increasing SES scores; it was also more often reported by men with hunger in their households, but this was not significant in multivariable modeling. There were no differences in victimization among men who had ever had sex with a woman, who had married a woman, or who had a current female partner. In bivariate analyses, men with current male partners or both current male and female partners were more likely to report victimization, but these associations were nonsignificant in multivariable analyses.

**Table 2:** Socio-demographic characteristics, sexual behavior, and sexual attraction by lifetime history of male-on-male sexual violence victimization.

Category	Characteristic	SV Victims (*n* = 162)		Non-Victims (*n* = 1,526)		*p*-Value	Odds Ratio (95% CI)	aOR^a^ (95% CI)
		Percent (Number)	95% CI	Percent (Number)	95% CI			
**Demographics**	**Racial group**							
	Non-black African	9.4 (15)	(5.4–15.7)	15.7 (240)	(11.9–20.5)	**0.02**	Ref	Ref
	Black African	90.6 (145)	(84.3–94.6)	84.3 (1,286)	(79.5–88.1)		**1.85 (1.03–3.30)**	**2.06 (1.04–4.10)**
	**Age group**							
	18–24 y	34.6 (56)	(48.7–54.7)	53.5 (817)	(50.4–56.6)	**<0.001**	Ref	Ref
	25 y and older	65.4 (106)	(57.2–72.9)	46.5 (709)	(43.4–49.6)		**2.18 (1.54–3.08)**	**1.68 (1.08–2.61)**
	**Secondary education**	41.0 (66)	(33.0–49.5)	40.5 (615)	(37.5–43.6)	0.91	1.00 (0.71–1.41)	
**SES**	Mean SES score (range: 6–19)	11.7 (n/a)	(11.3–12.2)	12.6 (n/a)	(12.5–12.8)	**<0.001**	**0.87 (0.81–0.93)**	**0.89 (0.82–0.96)**
	Hunger in household	65.5 (93)	(57.3–72.9)	50.3 (708)	(47.1–53.5)	**0.001**	**1.88 (1.30–2.72)**	
	Employed	60.4 (96)	(51.6–68.6)	54.0 (820)	(51.1–56.8)	0.15	1.32 (0.94–1.85)	
**Family and relationships**	Ever had sex with a woman	98.8 (160)	(95.2–99.7)	95.2 (1,449)	(93.8–96.3)	**0.04**	4.02 (0.97–16.7)	
	Ever married a woman	59.8 (95)	(51.7–67.3)	44.6 (671)	(41.7–47.4)	0.06	1.85 (1.32–2.59)	
	Ever fathered children	63.4 (102)	(55.8–70.4)	44.0 (657)	(41.1–46.9)	**<0.001**	**2.20 (1.56–3.11)**	**1.68 (1.09–260)**
	Current female partner	79.9 (127)	(73.1–85.3)	76.4 (1,158)	(73.6–79.0)	0.33	1.22 (0.73–2.06)	
	Current male partner	5.6 (9)	(3.0–10.0)	1.2 (18)	(0.8–1.8)	**<0.001**	**4.94 (2.11–11.6)**	
	Current male and female partners (both)	4.4 (7)	(2.1–8.9)	1.0 (15)	(0.6–1.6)	**<0.001**	**4.93 (1.90–12.8)**	
**Sexual attraction**	Women	82.7 (134)	(76.0–87.8)	86.6 (1,313)	(84.7–88.3)	**<0.001**	Ref	
	Men/both	14.8 (24)	(10.1–21.3)	6.8 (103)	(5.6–8.2)		**2.29 (1.40–3.75)**	
	Not sure	2.5 (4)	(0.9–6.5)	6.7 (101)	(5.4–8.1)		0.39 (0.14–1.09)	
**Other male–male experiences**	Consensual male–male sex	19.9 (32)	(14.7–26.3)	4.0 (61)	(3.1–5.2)	**<0.001**	**6.16 (3.79–10.0)**	**7.34 (4.30–12.5)**
	Male SV perpetrator	5.8 (9)	(3.1–10.6)	2.7 (41)	(2.0–3.7)	**0.02**	**2.20 (1.03–4.72)**	

Values in bold are significant at *p*<0.05.

a Only correlates significant at *p*<0.05 presented.

n/a, not applicable; Ref, reference value; SV, sexual violence.

### Prevalence and Socio-Demographic Correlates of Male-on-Male Sexual Violence Perpetration

Overall, 2.9% (*n* = 50; 95% CI 2.1–3.8) of participants reported any perpetration of male-on-male sexual violence, and 1.2% (*n* = 31; 95% CI 1.2–2.5) had perpetrated oral/anal male rape. There was no difference in the reported prevalence of perpetration by age, race, or SES (Table 3). In bivariate analyses, men who had ever been married were more likely to report perpetration, while those with a current female partner were less likely to report this. Men with a current male partner and men with both male and female partners were more likely to report having perpetrated sexual violence against men. However, these associations were nonsignificant in multivariable analyses. In the adjusted analyses, men with secondary school education or higher and men reporting hunger in their households were significantly more likely to report perpetration, while perpetration was less often reported by men with a primary sexual attraction to women.

**Table 3:** Socio-demographics, sexual behavior, and sexual attraction by lifetime history of male–male sexual violence perpetration.

Category	Characteristic	SV Perpetrators (*n* = 50)		Non-Perpetrators (*n* = 1,623)		*p*-Value	Odds Ratio (95% CI)	aOR^a^ (95% CI)
		Percent (Number)	95% CI	Percent (Number)	95% CI			
**Demographics**	Racial group							
	Non-black African	20.0 (10)	(10.4–35.1)	14.7 (239)	(11.1–19.3)	0.33	Ref	
	Black African	80.0 (40)	(64.9–89.6)	85.3 (1,382)	(80.7–88.9)		0.67 (0.30–1.48)	
	**Age group**							
	18–24 y	54.0 (27)	(41.6–65.9)	51.5 (836)	(48.4–54.6)	0.70	Ref	
	25 y and older	46.0 (23)	(34.1–58.4)	48.5 (787)	(45.4–51.6)		0.94 (0.52–1.69)	
	**Secondary education**	46.9 (23)	(33.0–61.4)	40.1 (648)	(37.1–43.2)	0.35	1.43 (0.78–2.61)	**2.05 (1.04–4.03)**
**SES**	Mean SES score (range: 6–19)	12.2 (n/a)	(11.5–13.0)	12.6 (n/a)	(12.4–12.7)	0.44	0.95 (0.85–1.07)	
	Hunger in household	70.2 (33)	(57.3–80.6)	51.3 (773)	(48.2–54.4)	**0.005**	**2.55 (1.30–5.01)**	**2.15 (1.05–4.38)**
	Employed	56.0	(41.7–69.4)	54.4	(51.6–57.2)	0.82	1.08 (0.60–1.95)	
**Family and relationships**	Ever had sex with a woman	98.0 (49)	(87.2–99.7)	95.5 (1,543)	(94.3–96.6)	0.40	2.32 (0.31–17.6)	
	Ever married a woman	64.0 (32)	(50.7–75.4)	44.9 (720)	(42.1–47.8)	0.58	**2.20 (1.20–4.03)**	
	Ever fathered children	53.1 (26)	(39.6–66.1)	45.5 (725)	(42.9–48.3)	0.28	1.35 (0.75–2.41)	
	Current female partner	71.4 (35)	(59.5–81.0)	87.0 (1,403)	(85.1–88.8)	**<0.001**	**0.35 (0.18– 0.70)**	
	Current male partner	12.0 (6)	(5.7–23.7)	1.2 (20)	(0.8–1.9)	**<0.001**	**11.0 (3.80–32.0)**	
	Current male and female partners (both)	8.2 (4)	(3.2–19.5)	1.1 (18)	(0.7–1.7)	**<0.001**	**7.74 (2.20–27.2)**	
**Sexual attraction**	Women	60.0 (30)	(44.2–74.0)	87.0 (1,402)	(85.2–88.6)	**<0.001**	Ref	Ref
	Men/both	26.0 (7)	(15.1–40.9)	7.0 (76)	(5.8–8.4)		**5.69 (2.75–11.8)**	**4.18 (1.81–9.70)**
	Not sure	14.0 (7)	(7.3–25.3)	6.0 (97)	(4.9–7.4)		**3.50 (1.42–8.64)**	**3.36 (1.28–8.83)**
**Other male–male experiences**	Consensual male–male sex	20.0 (10)	(11.6–32.4)	5.1 (82)	(4.2–6.3)	**<0.001**	**5.02 (2.27–11.1)**	**3.10 (1.22–7.90)**
	Male SV victim	18.0 (9)	(10.5–29.2)	9.1 (146)	(7.7–10.7)	**0.02**	**2.16 (1.00–4.72)**	

Values in bold are significant at *p*<0.05.

a Only correlates significant at *p*<0.05 presented.

n/a, not applicable; Ref, reference value; SV, sexual violence.

### Correlations between Consensual Male–Male Sexual Behavior and Male-on-Male Sexual Violence

Men with a history of consensual male–male sexual behavior were over seven times more likely than other men to report sexual violence victimization (aOR = 7.34; 95% CI 4.30–12.5) after controlling for other demographic correlates associated with victimization (Table 2). MSM were also more likely to report perpetration of male-on-male sexual violence (aOR = 3.10; 95% CI 1.22–7.90) (Table 3). Framed another way, both victims (aOR = 6.92; 95% CI 4.11–11.6) and perpetrators (aOR = 2.47; 95% CI 1.04–5.87) of male-on-male sexual violence were more likely to report having had consensual sex with a man, after adjusting for socio-demographic correlates (Table 1). In contrast, while violence victimization and violence perpetration were significantly correlated in bivariate analyses, these correlations were nonsignificant in multiple regression models (see Tables 2 and 3). MSM victims of sexual violence also reported more severe violence: among men reporting sexual violence victimization, MSM were more likely than non-MSM (men reporting no consensual male–male sexual behavior) to report being raped: 62.5% (*n* = 20/32) of MSM victims of sexual violence reported oral or anal rape, compared to only 28.7% (*n* = 37/129) of non-MSM victims; *p*<0.0001.

### Correlations with HIV Serostatus

In general, men reporting no consensual male–male sexual behavior, no sexual violence victimization, and no sexual violence perpetration had the lowest HIV prevalence, at 17.0% (Table 4). The highest prevalence of HIV infection occurred among men reporting consensual oral/anal sex (31.4%) and men reporting oral/anal rape perpetration (34.8%). There was no difference in HIV prevalence among men with and without a current male sexual partner (19.1% versus 28.3%, *p* = 0.41), irrespective of lifetime history of consensual oral/anal sex.

**Table 4:** Prevalence of HIV infection by lifetime history of consensual male–male sex, male-on-male sexual violence victimization, and male-on-male sexual violence perpetration.

Group	Category of Potential Exposure to HIV through Male–Male Contact	Percent (Number) HIV+	95% CI
**No male–male contact (consensual or nonconsensual)**	No male-on-male contact (consensual or nonconsensual) (*n* = 992)	17.0 (170)	14.2–20.2
	No oral/anal contact (consensual or nonconsensual) (*n* = 1,148)	18.1 (206)	15.5–21.2
**By type of male–male contact** a	**Consensual male–male sexual contact**		
	Any consensual male–male sexual behavior (*n* = 73)	27.4 (20)	17.6–40.0
	Consensual oral/anal male–male sexual behavior (*n* = 35)	31.4 (11)	18.0–48.9
	**Sexual violence victimization**		
	Any male-on-male victimization (*n* = 131)	23.7 (31)	16.3–33.1
	Oral/anal male-on-male victimization (*n* = 46)	17.4 (8)	8.9–31.1
	**Sexual violence perpetration**		
	Any male-on-male perpetration (*n* = 36)	27.8 (10)	17.3–41.5
	Oral/anal male-on-male perpetration (*n* = 16)	34.8 (6)	20.2–52.9

a Participants reporting multiple different types of contact may be classified within more than one of these groups.

In multivariable regression modeling (Table 5), men who reported perpetrating any type of male-on-male sexual violence were more likely than men who had not to be HIV+ (aOR = 2.55; 95% CI 1.02–6.38). Men reporting oral/anal sex with a man were more likely to be HIV+ than men with no such history (aOR = 3.11; 95% CI 1.24–7.80). Men reporting oral/anal rape perpetration were more likely to be HIV+ than non-perpetrators (aOR = 3.58; 95% CI 1.17–10.9). Victimization history was not associated with HIV status.

**Table 5:** Multivariable logistic regression models for prevalent HIV infection showing adjusted odds ratios for consensual male–male sex, sexual violence victimization, and sexual violence perpetration.

Model	Independent Variable	aOR	95% CI
**Model 1: including all types of experience**	Any consensual male–male sexual activity	1.62	0.80–3.24
	Any male–male sexual violence victimization	1.10	0.64–1.86
	Any male–male sexual violence perpetration	**2.55**	**1.02–6.38**
	**Other correlates of prevalent HIV**		
	Age >25 y	**7.33**	**4.89–11.0**
	Black	**4.79**	**2.34–9.78**
	Circumcised	**0.41**	**0.28–0.60**
	Hunger in household	**1.68**	**1.16–2.43**
**Model 2: including acts of oral/anal penetration only**	Consensual oral/anal male–male sex	**3.11**	**1.24–7.80**
	Rape victimization	0.74	0.29–1.89
	Rape perpetration	**3.58**	**1.17–10.9**
	**Other correlates of prevalent HIV**		
	Age >25 y	**7.49**	**5.00–11.2**
	Black	**4.93**	**2.41–10.1**
	Circumcised	**0.42**	**0.29–0.62**
	Hunger in household	**1.60**	**1.11–2.31**

Values in bold are significant at *p*<0.05.

## Discussion

In our population-based sample, approximately one in 20 men (5.4%) reported at least one lifetime occurrence of consensual sexual contact with a man, and nearly twice this proportion (9.6%) reported experience of male-on-male sexual violence victimization. MSM were over seven times more likely than other men to report male-on-male sexual violence victimization, and about three times more likely to report perpetration of such violence. Among the participants who provided blood for HIV testing, HIV prevalence was higher among men reporting a lifetime history of consensual oral/anal sex with a man, and also higher among men who had perpetrated male-on-male rape or other acts of male-on-male sexual violence. However, male rape survivors were not more likely than other men to be HIV+, a finding that parallels data among female rape survivors in South Africa [21],[22].

### Prevalence of Consensual Male–Male Sexual Behavior and Male–Female Concurrency

Our estimates of any consensual sexual activity between men, including consensual oral or anal sex, are consistent with reports from other developing countries [1],[4],[23], although we were unable to locate comparable population-based data from Africa. Oral sex was reported by 1.7% of men, and anal sex by 1.8%, in our sample. These figures may be low-end estimates, as 1.7% of those reporting consensual male–male sexual activity did not specify type of sexual act. Consistent with other settings [1],[4],[23], consensual male–male sexual behavior was associated with lower household SES. Because these data are cross-sectional, the extent to which MSM status leads to economic disadvantage and the extent to which economic disadvantage may motivate transactional encounters or relationships with other men is unknown, and will be important to explore in future research. Nonetheless, links between lower SES and consensual male–male sexual behavior need to be considered in planning service provision.

Men who reported consensual male–male sexual behavior did not differ from those reporting sex only with women in their lifetime sexual behavior with women, marriage history with women (same-sex marriage is legal in South Africa), or having a current female partner. These findings contrast sharply with those of Baral et al., garnered through venue-based sampling of self-identified MSM, who found that only 8% of MSM had a regular female partner and 18% identified as bisexual [24]. Our findings are closer to those of Lane et al. and Sandfort et al., who found that over half of self-identified MSM had had a female partner in the last 6 mo [12],[13]. They are also closer to the common accounts of male–male sexual practices in, for example, mine hostels and prisons, where lengthy same-sex relationships have been common among men who self-identify as heterosexual and prefer hetero sex in other contexts [25]–[27]. Most of the men in our sample who reported consensual male–male sexual behavior currently had a female partner, and stated a primary sexual attraction to women. Among the small group of men in our sample with current male partners, 80% (*n* = 21/26) also had current female partners. Having a current male partner is a crude indicator of recent male–male sexual activity in our study, as men may have recently had sex with a man, but one who was not considered a partner, and there were no questions in the survey specifically about recent male–male sexual activity. We did not ask the men their self-identified sexual orientation (gay, bisexual, straight, etc.), so we cannot comment on behavior versus self-identification. Further research is needed to better understand the interplay between attraction, sexual identity, and behavior in southern Africa and to explore the overlaps between male–male and heterosexual sexual behavior, as well as the implications for HIV transmission risk and prevention.

The high population prevalence of male–female sexual concurrency reported here suggests that HIV prevention efforts must address men who have sex with both men and women and their male and female partners. It further suggests that the MSM who can be readily accessed through venues and social and sexual networks may represent a biased subset of the total population of MSM. Further population-based research using more standardized measures of current male–male sexual behavior and identity will be needed to confirm the proportions of MSM potentially reachable through targeted intervention. However, it seems likely that new strategies will be required to reach the full South African population of MSM for both research and prevention, and that it will be of benefit to mainstream MSM messaging in broader HIV prevention efforts.

### Male-on-Male Sexual Violence

Rethinking sexual health among men in South Africa must include addressing the high levels of male-on-male sexual violence and enabling victims to come forward for assistance from the police and health services. While male violence against women is rightly understood to be a public health crisis in South Africa because of its very high prevalence [20],[28]–[35] and established links with HIV infection [20]–[22],[31], male-on-male sexual violence in the general population has received little attention. The high levels of male-on-male victimization reported here are in keeping with those reported among male adolescents participating in an HIV prevention trial in part of the geographic area covered by this survey [17]. While this study affirms that male-on-male victimization is common among men regardless of sexual attractions, it also shows that MSM are far more likely than other men to experience sexual assault, and that MSM victims of assault are more likely than other victims to report oral or anal rape. Because these data are cross-sectional, it is impossible to determine which of these correlated behaviors/experiences occurred first in time or to make causal inference; nonetheless, it is clear that MSM report more frequent experience of male-on-male sexual violence victimization and perpetration than non-MSM. Indeed, the prevalence of rape victimization reported by MSM in this study is comparable to the prevalence of rape victimization reported by South African women [21],[22]. In addition, while the findings were nonsignificant in multivariable analyses (likely because of small cell sizes), the two-way associations between having a current male partner and both experiencing and perpetrating male-on-male sexual violence suggest the possibility that male-on-male intimate partner violence may also be a significant concern among MSM. This issue merits study in future research. Overall, while more research is needed to understand the risk factors and health consequences associated with male-on-male sexual violence in South Africa, it is clear that such violence deserves attention in HIV and sexual health programming, and in delivery of health, legal, and social services.

### Consensual Male–Male Sexual Behavior, Male-on-Male Sexual Violence, and HIV

Previous research from southern Africa on the prevalence of HIV among MSM has yielded mixed figures: 13.9% in a respondent-driven sampling survey in Soweto [12], 25.5% in a convenience sample of men in Cape Town [24], and 17.4% in a non-probability sample from Malawi, Namibia, and Botswana [9]. These findings have been compared to general population estimates of HIV prevalence from other data sources, with varying conclusions about whether MSM have a higher HIV prevalence. While the exposure variable we measured was lifetime male–male sexual history, our data allow direct comparison with non-MSM from the same sampling frame, and affirm that a lifetime history of oral/anal sex with men is associated with significantly higher HIV prevalence among men. Our data newly highlight that men who perpetrate sexual violence against other men are also more likely than non-perpetrators to be HIV+, affirming the need to include prevention of male-on-male sexual violence in a comprehensive HIV prevention strategy.

### Strengths and Limitations

A key strength of this study was the use of APDAs for data collection; these provided a totally private and anonymous environment for disclosure of illegal and stigmatized behavior. Social desirability bias is nonetheless a concern and may have led to underreporting or misclassification of all outcomes of interest. This study was not primarily designed to collect data regarding male–male sexual behavior, and the relevant questions were limited. In particular, we did not measure the frequency or timing of male–male sexual behavior, and so those actively engaged in MSM networks are pooled here with those who have had only one experience, which may not have been recent. Similarly, information was not collected on the situational contexts of experiences of sexual assault. Consequently, despite some hints in the data that point towards likely intimate partner violence, we are unable to distinguish categories of sexual violence (i.e., we cannot tell partner violence from non-partner violence or childhood sexual assault).

Self-completion of the questionnaire resulted in some missing data on some items. We did not retain information on the number of eligible men per household and so were not able to weight the analysis for this, but we have no reason to believe this would have made much difference to the estimates of association and are aware that it usually makes no difference to estimates [36],[37]. We recognize that population prevalence of various behaviors may differ in other parts of the country, but have no reason to believe the two provinces surveyed have a particular cluster of MSM (unlike the Western Cape, which is known for having highly visible gay male communities), nor an unusually high prevalence of violence. Participation rates for the overall survey were 76% among enumerated and eligible households, but estimated at only 62% of potentially eligible households; additionally, only 70% of men completing the survey also provided blood samples for HIV testing. This may have introduced participation bias and therefore tempers the potential generalizability of the findings, particularly with regard to HIV. We note, however, that participation in our survey, including the HIV component, is on par with participation rates obtained in similar best-practice household-based surveys in South Africa [38]–[40]. Finally, despite the large sample size, the absolute numbers of MSM and survivors and perpetrators of male-on-male sexual violence in this study were fairly small, which impacts the precision of our estimates. Nonetheless, our method allows direct comparison of MSM and men who have experienced male-on-male sexual violence victimization and/or perpetration to other men drawn from the general population sample, and thereby offers improved generalizability compared to many sampling methods used to target hidden populations.

### Conclusion

In this population-based household survey of adult South African men, approximately one in 20 men reported consensual sexual contact with a man, while approximately one in ten reported being sexually assaulted by another man, and around 3% reported perpetrating such assault. These data emerge from one of the first African datasets to directly compare MSM and non-MSM from the same sampling frame, as well as one of the first to link male-on-male sexual violence with HIV serostatus. HIV prevalence was significantly higher among men reporting a lifetime history of consensual penetrative sex with men and among men who had sexually assaulted other men than it was among men in the general population. Male–female concurrency was common among MSM in these data, suggesting that prevention messaging about the risks associated with male–male sex needs to be mainstreamed into HIV prevention messaging for the general population in a way that does not invite homophobic stigmatization. Also required are further efforts to promote access to post-rape services for male survivors of sexual violence. These interventions will be effective and accessible only if they are provided in a context of active efforts to destigmatize lesbian, gay, bisexual, and transgender (LGBT) identities, and of active enforcement of South Africa's constitutional protections against anti-LGBT discrimination and in support of marriage equality. While this work offers important insight into the sexual health needs of South African men, it requires replication in other African countries, where decriminalization of consensual homosexual behavior will be a prerequisite for the broad success of any public health research or intervention.

## Abbreviations

aOR: adjusted odds ratio
APDA: audio-enhanced personal digital assistant
EA: enumeration area
MSM: men who have sex with men
SES: socioeconomic status
